# Low seasonal influenza vaccine uptake and related 7C-attitudes among swine farm managers in Brittany, France, 2023–2024: a cross-sectional survey

**DOI:** 10.1016/j.onehlt.2026.101420

**Published:** 2026-04-19

**Authors:** Asma Haddad, Jonathan Roux, Anne-Laure Maillard, Laurie Detrimont, Bertrand Gagnière, Claudio Trombani, Judith E. Mueller, Pascal Crépey

**Affiliations:** a University of Rennes, EHESP, CNRS, Inserm, Arènes–UMR 6051, RSMS–U 1309, Rennes, France; b Santé publique France, Saint-Maurice, France; c Organisation Sanitaire Porc (OS Porc) Bretagne, Rennes, France; d Breizhpig SCOP SAS, Plouédern, France; e Institut Pasteur, Paris Cité University, Emerging Disease Epidemiology Unit, Paris, France

**Keywords:** Vaccination, Seasonal influenza, One Health, Swine farm, Vaccine uptake, 7C psychological antecedents of vaccination, Vaccine willingness

## Abstract

**Background:**

Swine farmers are at increased risk of zoonotic influenza virus transmission. In France, seasonal influenza vaccination is recommended for swine farmers, yet little is known about vaccine uptake in this at-risk group.

**Objectives:**

To assess seasonal influenza vaccination uptake, its determinants, and associated attitudinal factors among swine farm managers in Brittany during the 2023–2024 influenza season.

**Methods:**

A cross-sectional survey was conducted among a random sample of swine farm managers. Data were collected using a standardized questionnaire, including attitude items according to the French 7C-model of psychological antecedents of vaccination.

**Results:**

Among 735 respondents, 14.0% [11.8%–16.4%] declared they have been vaccinated for the 2023–2024 season, and 35.0% [32.1%–38.0%] were unaware of the national recommendation. Only 33.5% demonstrated a knowledge score above 2/4. Vaccine uptake was significantly associated with older age, prior severe influenza (OR: 3.29 [1.97–5.50]), and receiving a vaccination voucher from the agricultural social security system (OR: 2.76 [1.66–4.59]). However, only 31.8% [28.6%–34.8%] reported having received one. Five among the seven components of the 7C-model were associated with vaccine uptake.

Unvaccinated unwilling to vaccinate were characterised by low collective responsibility (R^2^ = 16.98%) and unfavourable benefit-risk calculation (R^2^ = 16.84%), while unvaccinated willing to vaccinate were mainly influenced by vaccine accessibility (R^2^ = 17.32%) and negative social conformism (17.20%).

**Conclusion:**

Seasonal influenza vaccination uptake among swine farm managers is low. Targeting this population through tailored strategies is essential. These findings are relevant nationally and internationally and can inform One Health coordinated prevention efforts for professionals exposed to zoonotic influenza viruses.

## Highlights

• Low influenza vaccination uptake (14%) among swine farm managers in Brittany
• Individual, programmatic, and social factors shape vaccination behaviour
• Two unvaccinated profiles identified, those willing to vaccinate and those unwilling, with distinct 7C-attitude patterns
• Actionable One Health strategies needed for occupational influenza vaccination
• Need tailored communication, on-site vaccines, and veterinarians involvement in promoting vaccination and preventive health.

## 1 Introduction

Influenza represents a persistent pandemic threat due to its capacity for interspecies transmission and genetic reassortment. The One Health approach underscores the importance of monitoring influenza at the human–animal interface, a critical point in zoonotic transmission. Historically, swine have been recognized as the primary “mixing vessel” for the emergence of new influenza viruses through genetic recombination [1].

Vaccination is the most effective means of preventing and controlling seasonal influenza, reducing both disease incidence and severity  [2], [3]. Vaccination strategies vary by country. In Europe, most countries, including France, recommend seasonal influenza vaccination (SIV) for people at high risk of complications [4]. Influenza vaccination for professionals exposed to swine and avian influenza viruses has been recommended by the French National Authority for Health (HAS) since 2022 as an essential collective protection measure and was subsequently included in the national vaccination schedule [5], [6].

France, Europe's third-largest pork producer, exports 30% of its production. [7]. The sector is particularly concentrated in the Brittany region with over 4000 farms totalling 6.8 million swine in 2022—more than half (56%) of the national herd [7]. In France, influenza poses a significant challenge to swine farms, where it is widespread and persistent, without a discernible seasonal pattern [8]. Despite its obvious importance, little is known about SIV among swine farmers. We conducted a cross-sectional study among swine farm managers in Brittany following the 2023–2024 influenza season to assess SIV uptake, and the determinants and attitudinal factors associated with vaccination. This study is framed within the French 7C-model of psychological antecedents of vaccination [9], [10], [11], which explores key attitudinal determinants of vaccination.

## 2 Methods

### 2.1 Survey organization

A cross-sectional survey was conducted in a single day by 38 trainees from a field epidemiology training course (IDEA 2024), held at the French School of Public Health (EHESP). A random sample of swine farms was selected by the French sanitary organization overseeing pig health and biosecurity, *Organisation Sanitaire Porc (OS Porc)* Brittany, using data from BDPORC, a database listing swine farms. The sampling ensured representativeness regarding the geographical *départements* of Brittany (Côtes-d'Armor, Finistère, Ille-et-Vilaine, Morbihan), farm size (small, average, large or multisite where different sizes for each stage of pig rearing is registered), and type of farming activity (with and without sows or multisite where different stages of pig rearing are carried out).

### 2.2 Data collection

Data were collected through telephone interviews using a standardized questionnaire, which was developed and completed via LimeSurvey 6.4.8 (LimeSurvey Community Edition, Hamburg, Germany) (Supplementary Material S1). The questionnaire covered participants' characteristics, as well as their information and knowledge about influenza and its prevention. We assessed institutional and programmatic determinants such as having received a vaccination voucher. Attitudinal factors were assessed using the 7C-model of psychological antecedents (supplementary material S2).

Participants self-reported their seasonal influenza vaccine status for the 2023–2024 campaign. Unvaccinated individuals were asked about their willingness to receive the vaccine during the same season.

### 2.3 Knowledge and information

Knowledge was assessed using four items on influenza, its pandemic risk, and prevention, with a total score ranging from 0 to 4 (Supplementary Material S2).

In addition, we evaluated participants' awareness of the vaccination recommendation and the availability of free vaccination for professionals in contact with swine. We also explored satisfaction with the information received about influenza vaccination, as well as preferred sources of information.

### 2.4 Attitudinal factors

The questions on attitudinal factors were developed based on the French 7C-model of psychological antecedents of vaccination [9], [10], [11]. This model defines seven attitudinal dimensions (confidence in the vaccine, complacency, convenience, calculation, collective responsibility, social conformism and confidence in the system). In the present study, we explicitly relied on this French model, which extends the traditional 5C framework [12] by integrating two additional antecedents: social conformism and confidence in the system. The model assumes that the items need specific adaptation to each vaccination and target population. While the model has been developed and validated for vaccination against Covid-19 vaccination among healthcare workers and HPV vaccination among adolescents and parents, no questionnaire exists for influenza vaccination among farmers. We therefore formulated one item for each of the 7C dimensions to assess the corresponding attitude, except for social conformity and complacency, for which two items were developed each. For social conformism, we aimed to assess the influence of both personal and professional networks. Regarding complacency, we sought to evaluate both complacency towards influenza itself and the perceived need for vaccination, which are closely related. Items were evaluated on a 3-point Likert scale. Six components reflected vaccine-favourable attitudes, whereas high complacency represented a vaccine-unfavourable attitude. All items were formulated with a positive answer being vaccine-favourable except for confidence in the vaccine item, “I am afraid of the side effects of the flu vaccine”, which was reverse-coded to meet this criterion (R in Table 3). We also created binary 7C attitude variables opposing favourable to unfavourable or ‘don't know’ expressions.

### 2.5 Data analysis

The sample was stratified to account for the population structure using three variables: *département*, farm size, and type of farming activity. For descriptive statistics, categorical variables are presented as percentages with their 95% confidence intervals (95% CI), and continuous variables are reported as medians with interquartile ranges (IQR).

We conducted multivariable quasi-binomial logistic regressions to identify determinants of SIV uptake including key sociodemographic, farm-related, programmatic, and attitudinal factors (Supplementary Material S2). We evaluated the association between 7C attitudes (binary) and vaccine uptake (binary) with and without inclusion of sociodemographic, farm-related and programmatic variables. The same analysis keeping the 7C attitudinal factors in three categories is provided as supplementary material.

Willingness to be vaccinated was defined based on participants' answers to the question “Would you have liked to be vaccinated during the 2023–2024 campaign?”, grouping together those who responded yes, maybe, or don't know, and considering those who answered no as not willing to vaccinate.

To assess the psychological antecedents of vaccination, we computed scores, ranging from 0 to 1, for each of the seven dimensions of the 7C model. When two items were used for a given dimension, the score was calculated as the mean of the two binary values. We compared the mean scores for each 7C-attitude across three groups: vaccinated individuals, unvaccinated with and unvaccinated without expressed willingness to have been vaccinated in 2023–2024.

We then computed the coefficient of determination (pseudo-R^2^) from multivariable logistic regression models to assess the relative contribution of each psychosocial component to vaccine uptake and refusal. Two separate models were built for this, one contrasting vaccinated persons to unvaccinated without expressed willingness, and one contrasting them to unvaccinated with willingness. Each model included one of the 7C components as the primary explanatory variable, adjusted for age and sex. Higher pseudo-R^2^ values reflect a greater explanatory contribution of the corresponding component to lower vaccine uptake. To simplify matters, we refer to these values as R^2^ throughout the remainder of the article. Analyses were performed on the weighted sample using R version 4.4.2 [13].

## 3 Results

### 3.1 Participation and sample characteristics

Of the 3.370 farms registered in the *OS Porc* BDPORC database, 2.207 (65%) were included in the sampling frame. Of these, consent was obtained from 811 farms (69.3%), among which 735 farm managers completed the questionnaire (Supplementary fig. S1). Most respondents were male (87.8% [85.5%–89.8%]), with a median age of 51 years (IQR: 43–58), ranging from 23 to 91 years. A large proportion (81.9% [79.4%–84.3%]) reported having worked in contact with swine for over 15 years (Table 1).

**Table 1:** Influenza Vaccination Uptake Among Swine Farm managers: Socio-Demographic, experience, farm, and Knowledge Characteristics—Descriptive and Multivariable Analyses, Brittany, France (*n* = 735).

Variable^a^	Total sample *N* = 735 95% CI	Unvaccinated *n* = 633 95% CI	Vaccinated *n* = 102 95% CI	Bivariate analysis OR (95% CI, *p*-value)	Full multivariable model^b^ OR (95% CI, p-value)
Influenza vaccination in 2023–2024	14.0% [11.8%–16.4%]	-	-	-	-

Sex, %					
Female	12.2% [10.2%–14.5%]	12.3% [10.2%–14.8%]	11.4% [7.1%–18.0%]	–	–
Male	87.8% [85.5%–89.8%]	87.7% [85.2%–89.8%]	88.6% [82.0%–92.9%]	1.09 (0.61–1.93, *p* = 0.776)	1.39 (0.60–3.22, *p* = 0.439)

Age group, %					
(18,45]	32.1% [29.1%–35.2%]	35.4% [32.0%–38.9%]	11.7% [7.3%–18.4%]	–	–
(45,55]	33.8% [30.7%–37.0%]	32.9% [29.6%–36.4%]	39.3% [30.8%–48.4%]	3.59 (1.97–6.55, *p* < 0.001)	**4.67 (1.98–11.01, *p*** **<** **0.001)**
(55,65]	31.8% [28.8%–35.0%]	30.3% [27.1%–33.7%]	40.7% [32.3%–49.6%]	4.04 (2.24–7.27, p < 0.001)	**6.05 (2.46–14.86, p** **<** **0.001)**
(65,91]	2.3% [1.6%–3.4%]	1.4% [0.8%–2.4%]	8.3% [4.8%–14.0%]	18.23 (7.21–46.13, p < 0.001)	**40.73 (10.07–164.72, p** **<** **0.001)**

Education level, %					
Vocational certificate or lower	17.8% [15.4%–20.4%]	17.9% [15.4%–20.8%]	16.8% [11.2%–24.4%]	–	–
Baccalaureate	38.9% [35.7%–42.2%]	39.8% [36.4%–43.4%]	33.2% [25.2%–42.2%]	0.89 (0.50–1.57, *p* = 0.681)	1.02 (0.47–2.20, *p* = 0.960)
Baccalaureate +2 or higher	43.3% [40.1%–46.6%]	42.2% [38.8%–45.8%]	50.0% [41.2%–58.9%]	1.26 (0.74–2.15, *p* = 0.389)	1.85 (0.88–3.86, *p* = 0.103)

Years of experience, %					
< 15 years	18.1% [15.7%–20.6%]	19.7% [17.1%–22.5%]	8.2% [4.5%–14.4%]	0.36 (0.19–0.71, *p* = 0.003)	0.74 (0.30–1.83, *p* = 0.512)
≥ 15 years	81.9% [79.4%–84.3%]	80.3% [77.5%–82.9%]	91.8% [85.6%–95.5%]	–	–

*Département*, %					
Côtes-d'Armor	36.5% [34.4%–38.6%]	36.7% [34.2%–39.2%]	35.3% [27.4%–44.2%]	–	–
Finistère	23.4% [21.7%–25.2%]	21.3% [19.5%–23.3%]	36.0% [28.2%–44.6%]	1.75 (1.09–2.80, *p* = 0.020)	1.55 (0.84–2.84, *p* = 0.161)
Ille-et-Vilaine	21.9% [20.3%–23.7%]	22.6% [20.6%–24.7%]	18.1% [12.8%–25.0%]	0.84 (0.49–1.42, *p* = 0.510)	0.93 (0.49–1.73, *p* = 0.811)
Morbihan	18.2% [16.5%–19.9%]	19.4% [17.5%–21.5%]	10.6% [6.2%–17.7%]	0.57 (0.28–1.13, *p* = 0.107)	0.70 (0.30–1.65, *p* = 0.416)

Site size, %					
Small	15.7% [15.7%- 15.7%]	15.6% [14.8%- 16.5%]	16.4% [11.7%–22.4%	–	–
Average	22.8% [22.8%–22.8%]	22.3% [21.2%–23.4%]	25.7% [19.4%–33.1%]	1.10 (0.65–1.87, *p* = 0.723)	0.74 (0.37–1.49, *p* = 0.395)
Large	29.8% [29.8%–29.8%]	30.9% [29.7%32.0%]	22.9% [16.6%–30.5%]	0.71 (0.41–1.23, *p* = 0.221)	**0.43 (0.21–0.88, p** **=** **0.020)**
Multisite	31.7% [31.7%–31.7%]	31.2% [29.8%–32.6%]	35.1% [27.2%–43.9%]	1.08 (0.62–1.86, *p* = 0.794)	0.54 (0.27–1.11, *p* = 0.095)

History of human “severe” influenza, %					
No/don't know	72.3% [69.3%–75.2%]	75.7% [72.5%–78.6%]	51.1% [42.2%–60.0%]	–	–
Yes	27.7% [24.8%–30.7%]	24.3% [21.4%–27.5%]	48.9% [40.0%–57.8%]	2.99 (2.01–4.43, p < 0.001)	**3.29 (1.97–5.50, *p*** **<** **0.001)**

Influenza on the farm during the past six months, %					
No/don't know	74.4% [71.3%–77.2%]	75.7% [72.4%–78.6%]	66.7% [57.7%–74.6%]	-	-
Yes	25.6% [22.8%–28.7%]	24.3% [21.4%–27.6%]	33.3% [25.4%–42.3%]	1.55 (1.02–2.36, *p* = 0.042)	1.26 (0.75–2.12, *p* = 0.374)

Influenza vaccination in 2022, %					
No/don't know	83.2% [80.6%–85.6%]	94.3% [92.5%–95.7%]	14.9% [9.5%–22.5%]	–	–
Yes	16.8% [14.4%–19.4%]	5.7% [4.3%–7.5%]	85.1% [77.5%–90.5%]	94.87 (52.79–170.50, *p* < 0.001)	**–**

Knowledge of vaccination recommendation, %					
No	35.0% [32.1%–38.0%]	37.3% [34.1%–40.6%]	20.7% [14.8%–28.2%]	–	–
Yes	65.0% [62.0%–67.9%]	62.7% [59.4%–65.9%]	79.3% [71.8%–85.2%]	2.28 (1.47–3.54, *p* < 0.001)	1.76 (0.90–3.43, *p* = 0.098)

Declares having received a vaccination voucher, %					
No/don't know	68.4% [65.2%–71.4%]	72.6% [69.3%–75.7%]	42.1% [33.7%- 51.1%]	–	–
Yes	31.6% [28.6%–34.8%]	27.4% [24.3%–30.7%]	57.9% [48.9%–66.3%]	3.65 (2.46–5.42, p < 0.001)	**2.76 (1.66–4.59, *p*** **<** **0.001)**
Knowledge score; Mean (SD)	1.8 (1.2)	1.8 (1.2)	2.3 (1.3)	1.42 (1.19–1.68, *p* < 0.001)	0.97 (0.75–1.25, *p* = 0.817)

General vaccine adherence, %					
No	22.5% [19.9%–25.3%]	26.9% [23.9%–30.1%]	1.5% [0.5%–4.7%]	-	-
Yes	71.0% [68.0%–73.9%]	66.0% [62.6%–69.2%]	96.0% [91.7%–98.1%]	24.80 (7.64–80.51, p < 0.001)	–
Don't know	6.5% [5.0%–8.3%]	7.1% [5.5%–9.2%]	2.5% [0.9%–6.5%]	6.05 (1.29–28.30, *p* = 0.022)	–

CI: confidence interval; OR: odds ratio; SD: standard deviation.

a Note: Percentages are calculated based on the total number of valid responses for each variable.

b Odds ratio for being vaccinated in 2023–2024, from the full model including vaccinated colleagues, vaccinated entourage, reaction to vaccine incitation by OS Porc Brittany, justification for vaccination, fear of vaccination, felt protected by the vaccine, impact of the vaccine, and benefit/risk of vaccination.

### 3.2 Seasonal influenza vaccination uptake

**Fig. 1:**
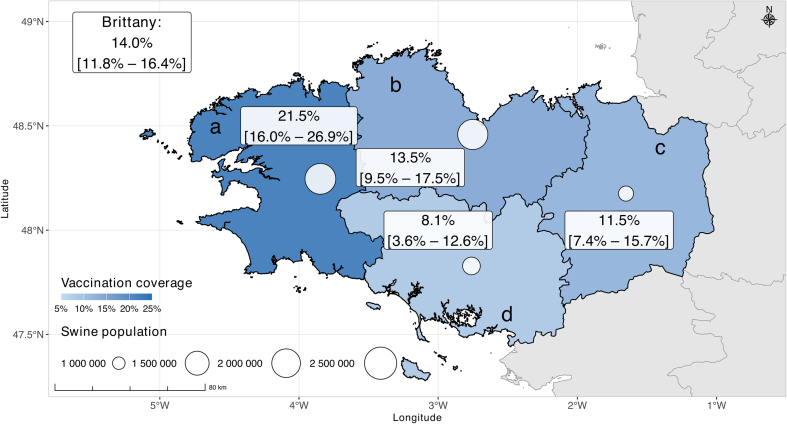
Reported influenza vaccination uptake (%) with 95% confidence intervals among swine farm managers and swine population by *département*, Brittany, France, 2023–2024 (a: Finistère, b: Côtes-d'Armor, c: Ille-et-Vilaine, d: Morbihan). Swine population data from Agreste, Annual Agricultural Statistics 2024 (provisional data) [14].

SIV uptake among Brittany swine farm managers during the 2023–2024 season was estimated at 14.0% [11.8%- 16.4%] (Table 1). Uptake varied by *département*, from 21.1% [16.0%; 26.9%] in Finistère to 8.1% [3.6%; 12.6%] in Morbihan (Fig. 1). More than a quarter of respondents (28% [25.5%; 31.6%]) reported that they would prefer to be vaccinated at a pharmacy, one-fifth (20% [17.4%; 22.8%]) preferred a general practitioner's office, 12% [10.0%; 14.4%] chose a nurse's office, and 9.2% [7.4%; 11.3%] preferred to be vaccinated at home.

For the 2022–2023 season, the estimated SIV uptake stood at 16.8% [14.4%–19.4%]. Among those vaccinated in 2023–2024, 85.0% [78.5%–91.5%] had also been vaccinated in 2022–2023. The decision of vaccine uptake was related to their professional activity for 39.8% [32.1%–48.0%] of respondents for these two seasons.

Among the 633 unvaccinated participants, 25.8% [22.5%–29.3%] expressed retrospective willingness to be vaccinated, while 74.2% (95% CI: 70.8–77.2) reported no willingness. The prevalence of these two groups within the total study population were 22.2% [19.5%–25.1%] and 63.8% [60.6%–67.0%], respectively.

### 3.3 Knowledge and information

Knowledge was highest regarding the transmission of the influenza virus from swine to humans (57.1% [53.8%–60.3%]) and lowest for viral reassortment between human and swine viruses with pandemic potential (39.4% [36.2%–42.7%]).

Overall, 16.8% [14.4%–19.4%] of the farm managers surveyed were unaware of any of the information presented, and only 33.5% had a knowledge score greater than 2 out of 4. Knowledge levels differed significantly between vaccinated and unvaccinated individuals for human-to-swine transmission, swine-to-human transmission, and pandemic viral reassortment (*p* < 0.001, p < 0.001, and *p* = 0.005, respectively), but not for flu preventive measures (*p* = 0.65).

Over one-third of respondents (35.0% [32.1%–38.0%]) were unaware of the HAS recommendation regarding influenza vaccination (Table 1), and nearly two-thirds (62.0% [58.6%–65.0%]) did not know that the vaccine is available free of charge for professionals working in swine farms.

Only 53% [49.5%–56.3%] reported being satisfied with the information they received this year about the influenza vaccination. Social media was cited as the primary source (29% [25.5%–32.0%]), followed by veterinarians (15% [12.2%–17.5%]) as preferred sources of information (Table 2).

**Table 2:** Knowledge and Information about Influenza and Influenza Vaccination among Swine Farm Managers: Descriptive Analysis, Brittany, France (*n* = 735).

Variable^a^	Total sample *N* = 735 95% CI	Unvaccinated *n* = 633 95% CI	Vaccinated *n* = 102 95% CI	p-value^b^
Knowledge of human-to-swine transmission, %				<0.001
Yes	51.2% [48.0%–54.5%]	48.9% [45.4%–52.4%]	65.4% [56.6%–73.4%]	
No	25.3% [22.5%–28.3%]	25.5% [22.5%–28.8%]	23.7% [16.8%–32.2%]	
Don't know	23.5% [20.9%–26.3%]	25.6% [22.6%–28.7%]	10.9% [6.6%–17.4%]	

Knowledge of swine-to-human transmission, %				<0.001
Yes	57.1% [53.8%–60.3%]	54.7% [51.2%–58.2%]	71.8% [63.3%–79.0%]	
No	22.1% [19.5%–24.9%]	22.7% [19.9%–25.7%]	18.3% [12.4%–26.2%]	
Don't know	20.8% [18.2%–23.6%]	22.6% [19.7%–25.7%]	9.9% [5.8%–16.4%]	

Knowledge of pandemic viral reassortment, %				0.005
Yes	39.4% [36.2%–42.7%]	37.4% [34.0%–40.9%]	52.2% [43.3%–61.0%]	
No	23.9% [21.2%–26.8%]	25.1% [22.1%–28.3%]	16.2% [10.7%–23.7%]	
Don't know	36.7% [33.6%–39.9%]	37.5% [34.1%–41.0%]	31.6% [24.0%–40.3%]	

Knowledge of flu preventive measures, %				0.65
Yes	48.2% [44.9%–51.5%]	48.2% [44.7%–51.8%]	48.0% [39.2%–56.9%]	
No	41.3% [38.1%–44.6%]	40.9% [37.5%–44.5%]	43.7% [35.1%–52.7%]	
Don't know	10.5% [8.6%–12.7%]	10.8% [8.8%–13.3%]	8.3% [4.7%–14.4%]	

Knowledge score, %				<0.001
0	16.8% [14.4%–19.4%]	18.4% [15.8%–21.4%]	6.3% [3.0%–12.7%]	
1	21.6% [19.0%–24.4%]	20.7% [18.0%–23.7%]	26.9% [19.8%–35.3%]	
2	28.2% [25.3%–31.3%]	29.8% [26.7%–33.2%]	18.1% [12.4%–25.6%]	
3	23.1% [20.4%–26.0%]	22.8% [19.9%–25.9%]	24.7% [17.7%–33.4%]	
4	10.4% [8.5%–12.7%]	8.2% [6.4%–10.4%]	24.0% [17.1%–32.6%]	

Awareness that the vaccine is free of charge, %				<0.001
Yes	38.0% [35.0%–41.4%]	35.5% [32.2%–39.0%]	53.4% [44.5%–62.2%]	
No/don't know	62.0% [58.6%–65.0%]	64.5% [61.0%–67.8%]	46.6% [37.8%–55.5%]	

Satisfaction with received information, %				<0.001
Satisfied	53.0% [49.5%–56.3%]	50.0% [46.3%–53.7%]	71.1% [62.1%–78.7%]	
Unsatisfied	31.0% [27.6%–33.9%]	31.6% [28.3%–35.1%]	25.0% [17.8%–33.8%]	
Neutral	16.0% [14.0%–19.1%]	18.4% [15.7%–21.4%]	3.9% [1.6%–9.3%]	

Preferred sources of information, %				0.001
TV/radio	1.1% [0.5%–2.1%]	1.1% [0.5%–2.4%]	0.8% [0.1%–3.8%]	
Health professionals	6.0% [4.5%–7.8%]	6.0% [4.4%–8.0%]	6.0% [2.9%–11.9%]	
Specialized press	8.9% [7.0%–11.3%]	9.7% [7.5%–12.3%]	4.8% [2.2%–10.2%]	
Professional associations	11.0% [8.9%–13.3%]	10.3% [8.2%–12.9%]	14.1% [8.7%–22.0%]	
Cooperative technician	8.9% [7.0%–11.1%]	9.8% [7.8%–12.4%]	3.1% [1.1%–8.3%]	
Uniporc (Interprofessional body)	2.6% [1.6%–4.1%]	2.2% [1.3%–3.7%]	4.9% [2.0%–11.6%]	
OS Porc Bretagne	0.6% [0.2%–1.3%]	0.7% [0.3%–1.6%]	0.0% [0.0%–0.0%]	
Veterinarians	**15% [12.2%–17.5%]**	13.4% [10.9%–16.4%]	21.5% [14.5%–30.7%]	
SMS (text messages)	5.4% [3.9%- 7.3%]	6.2% [4.5%–8.4%]	0.8% [0.2%–4.1%]	
Email	8.3% [6.5%–10.7%]	9.3% [7.2%–12.0%]	2.6% [0.7%–8.8%]	
MSA	0.5% [0.2%–1.3%]	0.6% [0.2%–1.6%]	0.0% [0.0%–0.0%]	
Social media	**29.0% [25.5%–32.0%]**	26.8% [23.5%–30.4%]	39.5% [30.6%–49.1%]	
None	3.6% [2.4%–5.2%]	3.9% [2.6%–5.7%]	1.9% [0.3%–10.7%]	

CI = Confidence Interval.

a Note: Percentages are calculated based on the total number of valid responses for each variable.

b Pearson's X^2^ test with Rao & Scott adjustment.

### 3.4 Determinants of influenza vaccination

In the multivariable logistic regression analysis, older age was significantly associated with influenza vaccine uptake in 2023–2024. A strong age-related gradient was observed. Compared with the youngest age group (18–45 years), participants aged 45–55, 55–65, and 65–91 years had progressively higher odds (OR = 4.67 [1.98–11.01], OR = 6.05 [2.46–14.86], and OR = 40.73 [10.07–164.72], respectively). Similarly, a self-reported history of “severe” influenza was associated with higher odds of SIV in 2023–2024 (OR: 3.29 [1.97–5.50]) (Table 1). Receiving a voucher for SIV coverage from the *Mutualité Sociale Agricole* (MSA), the French social security organization dedicated to farmers and agricultural workers, was also positively associated with vaccination (OR: 2.76 [1.66–4.59]). However, only 31.6% [28.6%–34.8%] of farm managers reported having received one.

### 3.5 Attitudinal factors

In bivariate models, general vaccine adherence among pig farm managers and SIV in 2022 showed strong associations with current SIV (OR: 24.80 [7.64–80.51], OR: 94.87 [52.79–170.50], respectively). To remove their masking effect due to their association with more precise attitudinal factors, we decided to remove these variables from the full model (Table 1). In the full multivariable model, perceiving more benefits than risks of vaccination was strongly associated with SIV in 2023–2024 (OR: 3.59 [1.52–8.46]). Similarly, the perception of vaccination as a collective action—reflecting the sense of collective responsibility—was significantly associated with vaccine uptake (OR: 2.69 [1.35–5.36]). Confidence in the vaccine, expressed by the absence of fear of side effects, was also associated with higher uptake (OR: 2.45 [1.34–4.45]). Social conformism was a significant predictor, with participants reporting vaccinated colleagues (OR: 2.68 [1.58–4.54]) or a vaccinated entourage (OR: 1.85 [1.04–3.31]) being more likely to be vaccinated. Finally, feeling protected by the vaccine—a marker of low complacency —was positively associated with SIV (OR: 2.41 [1.37–4.26]). Similar associations were found in the model restricted to the 7C psychological antecedents (Table 3).

**Table 3:** Influenza Vaccination Uptake Among Swine Farm Managers: The 7C Psychological Antecedents of Vaccination – Descriptive and Multivariable Analyses, Brittany, France (*n* = 735).

7C	Variable^a^	Total sample *N* = 735 95% CI	Unvaccinated *n* = 633 95% CI	Vaccinated *n* = 102 95% CI	Multivariable model with all 7C components OR^b^ (95% CI, *p*-value)	Fully adjusted multivariable model OR^c^ (95% CI, p-value)
**C1: Calculation**	Benefit/Risk of vaccination, %					
	“The flu vaccination provides more benefits than risks for me.”					
	No/Don't know	36.0% [32.9%–39.3%]	40.6% [37.1%- 44.2%]	7.8% [4.1%- 14.3%]	–	–
	Yes	64.0% [60.7%–67.1%]	59.4% [55.8%- 62.9%]	92.2% [85.6%- 95.9%]	**2.96 (1.37–6.38, *p*** **=** **0.006)**	**3.59 (1.52–8.46, *p*** **=** **0.004)**
**C2: Social Conformism**	Vaccinated colleagues, %					
	“Have people in your professional environment been vaccinated against the flu?”					
	No/Don't know	79.2% [76.3%- 81.8%]	83.2% [80.3%- 85.8%]	54.2% [45.2%- 62.9%]	-	-
	Yes	20.8% [18.2%- 23.7%]	16.8% [14.2%- 19.7%]	45.8% [37.1%- 54.8%]	**2.49 (1.57–3.94, p** **<** **0.001)**	**2.68 (1.58–4.54, *p*** **<** **0.001)**
	Vaccinated entourage, %					
	“Have people in your close circle been vaccinated against the flu?”					
	No/Don't know	40.5% [37.3%- 43.8%]	43.7% [40.2%- 47.3%]	20.7% [14.6%- 28.4%]	-	-
	Yes	59.5% [56.2%- 62.7%]	56.3% [52.7%; 59.8%]	79.3% [71.6%- 85.4%]	**1.88 (1.15–3.07, *p*** **=** **0.012)**	**1.85 (1.04–3.31, *p*** **=** **0.038)**
**C3: Collective responsibility**	Justification for vaccination, %					
	“Vaccination of swine farmers is justified to prevent the emergence of new flu viruses.”					
	No/Don't know	46.3% [43.0%- 49.7%]	51.2% [47.6%- 54.8%]	16.2% [11.0%- 23.2%]	-	-
	Yes	53.7% [50.3%- 57.0%]	Oui 48.8% [45.2%- 52.4%]	83.8% [76.8%- 89.0%	**2.61 (1.52–4.48, *p*** **=** **0.001)**	**2.69 (1.35–5.36, p** **=** **0.005)**
**C4: Confidence in the Vaccine**	Fear of vaccination, %					
	“I am afraid of the side effects of the flu vaccine” (R)					
	Yes/Don't know	39.8% [36.6%- 43.1%]	43.8% [40.3%- 47.4%]	15.4% [9.9%–23.0%]	-	-
	No	60.2% [56.9%- 63.4%]	56.2% [52.6%- 59.7%]	84.6% [77.0%- 90.1%]	**2.11 (1.21–3.69, *p*** **=** **0.009)**	**2.45 (1.34–4.45, p** **=** **0.004)**
**C5: Low Complacency**	Felt protected by the vaccine, %					
	“The flu vaccine provides good protection against the disease.”					
	No/Don't know	51.4% [48.1%- 54.7%]	55.8% [52.2%- 59.3%]	24.1% [17.2%- 32.8%]	-	-
	Yes	48.6% [45.3%- 51.9%]	44.2% [40.7%- 47.8%]	75.9% [67.2%- 82.8%]	**2.32 (1.42–3.79, p** **=** **0.001)**	**2.41 (1.37–4.26, *p*** **=** **0.002)**
	Impact of the disease, %					
	“I am concerned about the impact of flu outbreaks on my farm”					
	No/Don't know	44.3% [41.0%- 47.6%]	46.1% [42.5%- 49.6%]	33.0% [25.3%- 41.8%]	-	-
	Yes	55.7% [52.4%- 59.0%]	53.9% [50.4%- 57.5%]	67.0% [58.2%- 74.7%]	1.06 (0.67–1.67, *p* = 0.805)	1.12 (0.65–1.93, *p* = 0.685)
**C6: Confidence in systems**	Reaction to vaccine incitation by OS Porc Brittany, %					
	“If the OS Porc encourages you to get vaccinated against the flu, this would...”					
	Discourages me	3.4% [2.4%- 4.9%]	3.8% [2.6%- 5.4%]	1.3% [0.2%- 7.4%]	-	–
	Encourages me	36.4% [33.3% 39.7%]	32.7% [29.5%- 36.2%]	59.5% [50.5%- 67.9%]	2.23 (0.36–13.72, *p* = 0.387)	6.26 (0.74–52.59, *p* = 0.091)
	Have no effect on me	60.1% [56.8%- 63.3%]	63.5% [60.0%- 66.9%]	39.2% [30.9%- 48.2%]	1.70 (0.28–10.30, *p* = 0.566)	4.34 (0.50–37.28, *p* = 0.181)
**C7: Convenience**^d^	Easy access to vaccination, %					
	“In practice, is it easy for you to get vaccinated against the flu?”					
	No/Don't know	12.9% [10.9%- 15.3%]	15.0% [12.7%- 17.7%]	0.0% [0.0%- 0.0%]	–	–
	Yes	87.1% [84.7%- 89.1%]	85.0% [82.3%- 87.3%]	100.0% [100.0%- 100.0%]	–	–

CI: confidence interval; OR: odds ratio.

a Note: Percentages are calculated based on the total number of valid responses for each variable.

b Odds ratio for being vaccinated in 2023–2024 from the model including only the components of the 7C-model of psychological antecedents of vaccination.

c Odds ratio for being vaccinated in 2023–2024 from the model including age, sex, education level, years of experience, *département*, site size, influenza on the farm during the past six months, knowledge of vaccination recommendation, declaring having received a vaccination voucher, knowledge score and history of “severe” influenza.

d Odds ratio were not calculable due to the absence of vaccinated answering No or Don't know.

**Fig. 2:**
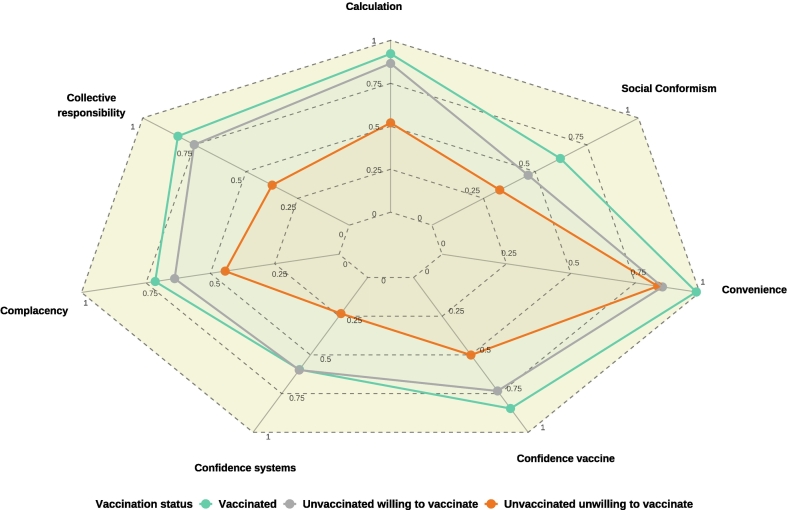
Comparison of the 7C attitudinal factors between vaccinated, unvaccinated willing and unvaccinated unwilling to vaccinate swine farm managers, Brittany, France, 2023–2024: mean scores, Red line: unvaccinated unwilling to vaccinate; Grey line: unvaccinated willing to vaccinate; Green line: vaccinated. (For interpretation of the references to colour in this figure legend, the reader is referred to the web version of this article.)

Across the 7C components, vaccinated individuals showed the highest mean scores, while the unvaccinated with willingness scored intermediately and closely resembled the vaccinated, except for perceived convenience. The unvaccinated unwilling had the lowest scores overall, though their convenience scores were similar to those willing to vaccinate (Fig. 2).

**Fig. 3:**
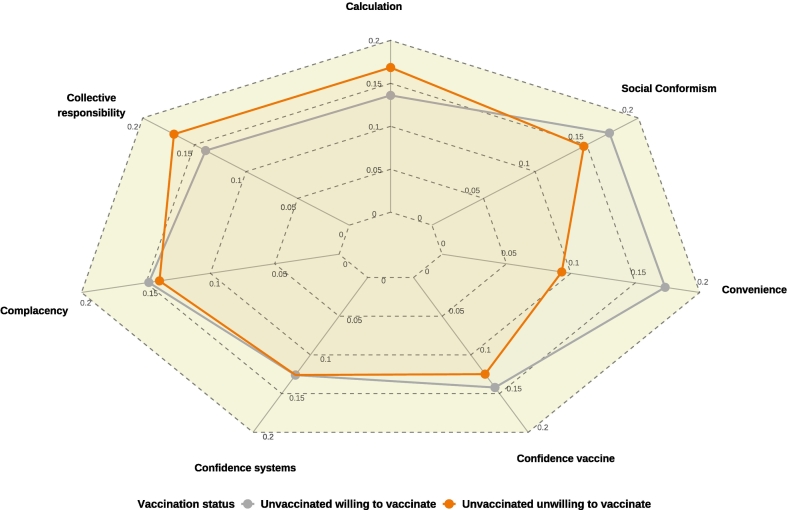
7C attitudinal factors for being unvaccinated among swine farm managers, Brittany, France, 2023–2024: coefficient of determination R^2^ of component-specific regression models, Red line: unvaccinated unwilling to vaccinate vs vaccinated; Grey line: unvaccinated willing to vaccinate vs vaccinated. (For interpretation of the references to colour in this figure legend, the reader is referred to the web version of this article.)

In regression models, lower uptake among the unwilling was mainly explained by collective responsibility (R^2^ = 16.98%) and benefit/risk calculation (R^2^ = 16.84%), whereas among the willing, convenience (R^2^ = 17.32%) and social conformism (R^2^ = 17.20%) were most influential (Fig. 3).

## 4 Discussion

Despite its recognized importance, the SIV uptake reported by swine farm managers in Brittany remains low. The estimated uptake during the 2023–2024 and 2022–2023 seasons were 14.0% [11.8%–16.4%] and 16.8% [14.4%–19.4%], respectively. These rates were higher than those reported among swine farm workers in Romania [15], likely because the present study focused specifically on swine farm managers, who may be more inclined to get vaccinated than workers. However, these coverages were lower than those observed in a comparable population in the United States [16]. The latter study was conducted during the 2009–2010 (H1N1 pandemic) and 2010–2011 influenza seasons, suggesting that workers may alter their vaccination behaviour based on their perceived risk.

The reported uptake remains well below the 75% target set by the World Health Organization and the HAS for at-risk populations. It is also lower than the coverage estimated for the same season among other French target groups. In 2023–2024, SIV coverage in these groups was estimated at 47.1%, with 54.0% among individuals aged 65 and over, and 25.4% among those under 65 considered at risk for severe influenza. These figures were lower than those reported for the 2022–2023 season (51.5% overall, 56.2% for those aged 65 and older, and 31.6% for those under 65 at risk) [17]. This downward trend was also observed in our study, with a decrease in self-reported coverage among swine farm managers between the 2022–2023 and 2023–2024 seasons. A similar trend was observed in a study conducted in France among healthcare professionals, showing insufficient coverage (22%) and a declining trend in SIV rates between the 2018–2019 and 2021–2022 seasons [18], [19].

General vaccine adherence among pig farm managers was 71%, lower than the national average of 83.7% reported in the 2023 Health Barometer [20], underscoring the need to explore vaccination behaviours in this specific context.

Older age and a history of prior SIV appear to be key determinants of SIV, consistent with previous studies conducted in France among healthcare workers [21]. Multiple studies have demonstrated that past behaviour strongly predicts current influenza vaccination uptake [22]. The association between older age and vaccine uptake has been well documented in both the general population and among healthcare professionals [23], [24]. This relationship may be explained by older adults' more frequent healthcare visits and past influenza experiences, which increase their willingness to receive vaccination recommendations, risk perception, and confidence in the vaccine [25], [26].

However, the absence of data regarding the health status of the study population, including whether they belong to risk groups for severe influenza, limits the interpretation of age-related effects in our context. Nonetheless, particular attention should be paid to professionals aged 65 years and older who are in contact with swine, as this group may be more vulnerable due to a higher prevalence of comorbidities and immunosenescence, which could reduce the effectiveness of influenza vaccines [27].

A history of having had the flu was associated with an increased likelihood of SIV. This supports previous findings in diverse populations, where prior experience of sickness has been linked to greater vaccine uptake [22].

In France, the influenza vaccine is fully reimbursed by the government for people targeted by vaccine recommendations, including professionals exposed to avian and swine influenza viruses. A voucher for full coverage is sent to them by the MSA, enabling them to obtain the vaccine free of charge. Nearly two-thirds were unaware that the influenza vaccine is free for swine farm professionals. Receipt of this voucher was strongly associated with vaccine uptake. However, only 31.6% [28.6%–34.8%] reported having received one. The financial aspect is frequently mentioned as a potential barrier to influenza vaccination and may contribute to suboptimal vaccine uptake [22]. The systematic issuance of vaccination vouchers and raising awareness that a voucher has been sent represent a priority avenue that should be further reinforced in the French context.

Knowledge levels and awareness regarding influenza and its prevention were low, with significant differences observed between vaccinated and unvaccinated individuals. Health literacy has a significant impact on the adoption of health-related behaviours and attitudes [28]. Efforts should target swine-exposed professionals, improving their health literacy on influenza, occupational risks, and vaccination guidelines and recommendations.

Communication via veterinarians remains a privileged and trusted channel for informing swine farmers. Veterinarians are regarded as the primary source of information on disease prevention and biosecurity for swine, cattle, and poultry farmers. Receiving more detailed explanations from the herd veterinarian considerably boosts farmers' interest in disease prevention [29]. Nonetheless, while social media are the most commonly cited source, they should be approached with caution: vaccine-related content is widely available, but distinguishing accurate from misleading information remains difficult [30].

Five components of the 7C-model emerged as significant predictors of vaccine uptake. Subsequent analysis of willingness to vaccinate revealed striking differences between two profiles, underscoring the relevance of the 7C-model for identifying differences between subgroups within a population, and guiding intervention design [10]. Individuals unwilling to vaccinate appear to be more independent and autonomous, primarily influenced by self-directed factors, including individual reasoning, and their own risk-benefit perception regarding vaccination. Conversely, individuals willing to vaccinate are mainly influenced by external, social, and contextual factors, such as social conformity and easy access to vaccination. They present a broadly similar profile compared to vaccinated individuals, suggesting that this population is highly mobilizable in favour of vaccination by acting on the pragmatic aspects of vaccination. Several studies conducted among healthcare professionals have shown that the lack of conveniently available vaccines is one of the main barriers to influenza vaccination [23]. Reaching out to the most isolated farmers and implementing targeted strategies, such as on-site vaccination initiatives and enhanced outreach through veterinarians, could significantly increase vaccine uptake among this population. Differentiating between these two profiles and targeting their specific needs could improve vaccination uptake.

This study has some limitations. The main ones are its cross-sectional design, and, as with most such surveys, the self-reported nature of the collected data, which may be subject to recall and desirability bias. Additionally, we did not collect data on individuals at high risk for severe influenza, which limits the interpretation of some findings.

Nevertheless, these limitations are balanced by several strengths. It is the first study to investigate SIV among professionals exposed to swine influenza viruses in France. It is based on a standardized questionnaire comprising a wide range of items with the integration of a theory-driven framework (the French 7C model). The study explores both vaccine uptake and willingness, along with their determinants. Furthermore, the use of stratified random sampling, the large sample size, and the high response rate are major strengths that enhance the sample's representativeness.

Our findings underscore the importance of translating the One Health approach into concrete vaccination strategies targeting occupationally exposed populations to prevent zoonotic transmission and foster coordination across human, animal, and environmental health sectors.

## 5 Conclusion

Our study provides an initial assessment of SIV uptake among swine farmers in Brittany, which remains low despite their increased occupational risk. Vaccine uptake appears to be a complex and multifaceted behaviour, shaped by individual, experiential, programmatic, and attitudinal factors. Using the French 7C-model, we identified two distinct profiles among unvaccinated individuals, based on their willingness to vaccinate: one group with an unfavourable perception of the vaccine's benefit-risk balance and low collective responsibility; the other primarily influenced by vaccine accessibility and social conformism. These findings highlight the need for tailored strategies that address the specific barriers associated with each profile. They contribute to a better understanding of influenza prevention among high-risk occupational groups and can inform more effective vaccination policies and interventions.

## Ethics considerations

Verbal informed consent was obtained from each participant. Data were anonymized, and the study followed the French Reference Methodology MR-004 for data protection standards. Ethical approval was not required under French law for this type of study. All necessary procedures related to this methodology—including notification and registration with the CNIL (French Data Protection Authority) and the Health Data Hub—have been carried out in coordination with the Data Protection Officer of the EHESP.

## CRediT authorship contribution statement

**Asma Haddad:** Writing – review & editing, Writing – original draft, Methodology, Investigation, Funding acquisition, Formal analysis, Data curation, Conceptualization. **Jonathan Roux:** Writing – review & editing, Formal analysis, Data curation. **Anne-Laure Maillard:** Methodology, Investigation, Conceptualization. **Laurie Detrimont:** Writing – review & editing, Methodology, Investigation, Conceptualization. **Bertrand Gagnière:** Writing – review & editing, Supervision, Methodology, Investigation, Conceptualization. **Claudio Trombani:** Writing – review & editing, Methodology, Conceptualization. **Judith E. Mueller:** Writing – review & editing, Supervision, Methodology, Investigation, Conceptualization. **Pascal Crépey:** Writing – review & editing, Visualization, Supervision, Resources, Project administration, Methodology, Investigation, Funding acquisition, Formal analysis, Data curation, Conceptualization.

## Funding sources

This work has benefited from an unrestricted grant from MSA. The funders had no role in study design, data collection and analysis, decision to publish, or preparation of the manuscript.

## Declaration of competing interest

PC declares consulting fees from Sanofi, AstraZeneca, Pfizer and Seqirus for work unrelated to this project. All other authors declare that they have no known competing financial interests or personal relationships that could have appeared to influence the work reported in this paper.

## Data Availability

Data will be made available on request.

## Appendix A Supplementary data

Questionnaire and additional methods
