# Supplementary material for: Low seasonal influenza vaccine uptake and related 7C-attitudes among swine farm managers in Brittany, France, 2023–2024: a cross-sectional survey
Source: One Health. 2026 Apr 19;22:101420. doi: 10.1016/j.onehlt.2026.101420 (PMC13129440; doi:10.1016/j.onehlt.2026.101420)

**Supplementary Material**

**Supplementary material S1. Questionnaire: original and English versions**

**French version**

1) Sexe: □ M □ F

2) Quel âge avez-vous? _____

3) Quel poste occupez-vous?

□ Chef d’exploitation        □ Responsable d’un secteur d’élevage

□ Technicien d’élevage     □ Agent d’élevage             □ Autre (préciser): ________

4) Quel est votre niveau d’étude?

□ Sans diplôme    □ Brevet     □ CAP–BEP      □ BAC Pro

□ BAC Technique/général   □ Bac +2 et plus    □ Sans réponse

5) Depuis combien d'années travaillez-vous en élevage porcin?  ___ ans

6) A quelle fréquence êtes-vous en contact avec des porcs? (Citer)

□ Au moins 1 fois /jour        □ Une ou plusieurs fois/ semaine

□ Moins d’une fois par semaine    □ Jamais

7) Avez-vous été vacciné contre la grippe cet hiver?

□ Oui        □ Non       □ Ne sait pas         □ Ne veut pas répondre

8) Si oui à la question 7, où avez-vous été vacciné?

□ Cabinet médical    □ Cabinet infirmier    □ Lieu de travail       □ Pharmacie

□ Domicile par un professionnel de santé   □ Domicile par moi-même  □ Autre

9) Si non à la question 7, auriez-vous souhaité être vacciné?

□ Oui     □ Non     □ Peut-être     □ Ne sait pas

10) Avez-vous été vacciné contre la grippe l’hiver précédent?

□ Oui      □ Non     □ Ne sait pas □ Ne veut pas répondre

11) Si réponse oui à la question 7 et/ou 10: Pour quelle raison?

□ En raison de votre activité professionnelle        □ Ne veut pas répondre

12) Pour les propositions suivantes, dites si vous êtes d’accord, ou pas d’accord :

|  | D’accord | Pas d’accord | Ne sait pas |
| --- | --- | --- | --- |
| Le virus de la grippe humaine peut se transmettre au porc |  |  |  |
| A l’inverse, le virus de la grippe porcine peut se transmettre à l’humain |  |  |  |
| Les virus de la grippe humaine et porcine peuvent se combiner et provoquer une pandémie |  |  |  |
| Le masque, les gants et l'hygiène des mains sont suffisants pour empêcher la transmission du virus de la grippe |  |  |  |

13) Savez-vous que la vaccination contre la grippe est recommandée pour toutes les personnes travaillant dans des élevages porcins ? □ Oui     □ Non

14) Le vaccin contre la grippe est-il gratuit pour les professionnels en contact avec les porcs ?  □ Oui     □ Non    □ Ne sait pas

15) Avez-vous déjà eu une grippe qui vous a empêché de travailler ?

 □ Oui     □ Non    □ Ne sait pas

16) Est-ce que des personnes de votre milieu professionnel ont été vaccinées contre la grippe?

  □ Oui     □ Non    □ Ne sait pas

17) Est-ce que des personnes de votre entourage proche ont été vaccinées contre la grippe?   □ Oui     □ Non    □ Ne sait pas

18) Pour les propositions suivantes, dites si vous êtes d’accord, ou pas d’accord :

|  | D’accord | P Pas d’accord | Ne sait pas |
| --- | --- | --- | --- |
| J Je suis favorable à la vaccination en général |  |  |  |
| La vaccination des éleveurs de porcs est justifiée pour prévenir l’apparition de nouveaux virus grippaux. |  |  |  |
| J’ai peur des effets indésirables du vaccin contre la grippe |  |  |  |
| Le vaccin contre la grippe protège bien contre la maladie |  |  |  |
| Je crains l’impact des épidémies de grippe dans mon élevage |  |  |  |
| La vaccination anti grippale a davantage de bénéfice que de risque pour moi |  |  |  |

19) Dans l’élevage où vous travaillez, y-a-t-il déjà eu de la grippe dans les 6 derniers mois?

□ Oui     □ Non    □ Ne sait pas

20) Si oui, S'agissait-il de grippes récurrentes?

 □ Oui     □ Non    □ Ne sait pas

21) Avez-vous reçu un bon de vaccination par la MSA?

 □ Oui     □ Non    □ Ne sait pas

22) En pratique, est-ce simple de vous faire vacciner contre la grippe?

□ Oui     □ Non    □ Ne sait pas

23) Quel est le meilleur moyen d’informer des professionnels, comme vous, sur la vaccination contre la grippe ?

□ TV /Radio                 □ Professionnels de santé      □ Presse spécialisée

□ Associations professionnelles    □ Technicien de la coopérative    □ Uniporc

□ OS Porc Bretagne    □ Vétérinaires        □ SMS         □ Email

□ MSA         □ Réseau sociaux           □ Aucun        □ Autre (préciser)____________

24) Où préférez-vous être vacciné contre la grippe?

□ Cabinet médical      □ Cabinet infirmier               □ Lieu de travail

□ Pharmacie        □ Autre       □ Pas de préférence     □ Je ne veux pas être vacciné

25) [Si lieu de travail], par qui souhaitez-vous être vacciné ?

□ Médecin           □ Infirmier   □ Vétérinaire       □ Autre ________

26) Si OS porc vous incite à vous faire vacciner contre la grippe, est ce que cela :(citer)

□ Vous encourage □ Vous dissuade    □ N’a pas d’impact sur vous   □ Autre ________

27) Etes-vous satisfait de l'information reçue cette année sur la vaccination antigrippale ?

 □ Oui     □ Non    □ Ne sait pas

28) Avez-vous des suggestions ou des remarques?

**English version**

1) Sex: □ M □ F

2) How old are you? _____

3) What is your position?

□ Farm manager        □ Livestock unit supervisor

□ Livestock technician     □ Livestock worker             □ Other (specify): ________

4) What is your level of education?

□ No diploma    □ Certificate (Brevet)       □ CAP – BEP (vocational certificate)                         □ Vocational baccalaureate                         □ Technical or general baccalaureate                   □ Post-secondary education (2+ years)      □ No response

5) How many years have you worked in swine farming?  __ years

6) How often are you in contact with pigs? (specify)

□ At least once per day          □ One or more times per week

□ Less than once per week    □ Never

7) Were you vaccinated against influenza this winter?

□ Yes        □ No       □ Don't know         □ Prefer not to say

8) If yes to question 7, where were you vaccinated?

□ Doctor's office    □ Nurse's office    □ Workplace       □ Pharmacy

□ At home by a healthcare professional   □ At home by myself □ Other

9) If no to question 7, Would you have liked to be vaccinated?

□ Yes  □ No  □ Maybe    □ Don’t know

10) Were you vaccinated against influenza the previous winter?

□ Yes     □ No     □ Don't know □ Prefer not to say

11) If yes to question 7 and/or 10: For what reason?

□ Because of your professional activity        □ Prefer not to say

12) For the following statements, indicate whether you agree or disagree:

| **Statement** | **Agree** | **Disagree** | **Don't know** |
| --- | --- | --- | --- |
| Human influenza virus can be transmitted to pigs |  |  |  |
| Conversely, swine influenza virus can be transmitted to humans |  |  |  |
| Human and swine influenza viruses can combine and cause a pandemic |  |  |  |
| Masks, gloves, and hand hygiene are sufficient to prevent influenza virus transmission |  |  |  |

13) Are you aware that influenza vaccination is recommended for all persons working in swine farms? □ Yes     □ No

14) Is the influenza vaccine free of charge for professionals in contact with pigs?

□ Yes     □ No    □ Don’t know

15) Have you ever had influenza that prevented you from working?

 □ Yes     □ No    □ Don’t know

16) Have people in your professional environment been vaccinated against influenza?

 □ Yes     □ No    □ Don’t know

17) Have people in your close circle been vaccinated against influenza?

□ Yes     □ No    □ Don’t know

18) For the following statements, indicate whether you agree or disagree:

| **Statement** | **Agree** | **Disagree** | **Don't know** |
| --- | --- | --- | --- |
| I am generally in favour of vaccination |  |  |  |
| Vaccinating swine farmers is justified to prevent the emergence of new influenza viruses |  |  |  |
| I am afraid of the side effects of the influenza vaccine |  |  |  |
| The influenza vaccine provides good protection against the disease |  |  |  |
| I am concerned about the impact of influenza outbreaks on my farm |  |  |  |
| For me, influenza vaccination has more benefits than risks |  |  |  |

19) Has there been influenza in the farm where you work in the past 6 months?

□ Yes     □ No    □ Don’t know

20) If yes to question 19, Were these recurrent influenza cases?

□ Yes     □ No    □ Don’t know

21) Did you receive a vaccination voucher from MSA?

□ Yes     □ No    □ Don’t know

22) In practice, is it easy for you to get vaccinated against influenza?

□ Yes     □ No    □ Don’t know

23) What is the best way to inform professionals like you about influenza vaccination?

□ TV / Radio                □ Healthcare professionals         □ Specialized press

□ Professional associations     □ Cooperative technician            □ Uniporc

□ OS Porc Bretagne                 □ Veterinarians                           □ SMS

□ Email                                     □ MSA                                        □ Social media

□ None                                     □ Other (specify): ____________

24) Where do you prefer to be vaccinated against influenza?

□ Doctor's office      □ Nurse's office                     □ Workplace

□ Pharmacy            □ Other       □ No preference     □ I do not want to be vaccinated

25) [If Workplace], By whom would you like to be vaccinated?

□ Doctor           □ Nurse       □ Veterinarian       □ Other ________

26) If the *OS Porc* encourages you to get vaccinated against influenza, would that :(citer)

□ Encourages you  □ Discourages you    □ Has no impact on you   □ Other ________

27) Are you satisfied with the information you received this year about influenza vaccination?

□ Yes     □ No    □ Don’t know

28) Do you have any suggestions or comments?

**Supplementary material S2. Methods (detailed)**

**Attitudinal factors**

The French 7C-model of psychological antecedents was developed from earlier models that initially included three, then five components [[1,2]](https://www.zotero.org/google-docs/?9Kkdw4). Initially, the five components assessed were: confidence in the vaccine, complacency, convenience, calculation, and collective responsibility. Confidence in the vaccine refers to the trust in the vaccine's safety and effectiveness, as well as the health authorities and officials who develop and license vaccines. Complacency occurs when the perceived risks of vaccine-preventable diseases are low, making vaccination seem unnecessary. Convenience is the opposite of constraints, which designates barriers—both structural and psychological—that hinder vaccination intention from becoming action. These may include availability, affordability and financial issues, geographical accessibility, time, immunization service, and health literacy. Calculation refers to the tendency to engage in extensive information searching, weighing perceived benefits and risks of vaccines. Collective responsibility is the willingness to protect others through one’s own vaccination by contributing to herd immunity.

Recently, a French model was developed where two additional psychological antecedents were evaluated. Confidence in systems reflects trust in the broader institutions involved in vaccination efforts, essentially authorities and employers. Social conformism refers to the influence of social norms and the vaccination stance of one’s peers. It is specifically this French model that we adopted as the conceptual basis for our investigation.

**Data analysis**

A knowledge score on influenza was developed based on four statements about the disease and its prevention methods. One point was awarded for each correct response to the following three statements: “Human influenza virus can be transmitted to swine,” “Conversely, swine influenza virus can be transmitted to humans,” and “Human and swine influenza viruses can combine and cause a pandemic.” An additional point was given for disagreeing with the statement: “Masks, gloves, and hand hygiene are sufficient to prevent influenza virus transmission.” The knowledge score ranged from 0 (no knowledge) to 4 (maximum knowledge).

The following variables were included as potential predictors of vaccination uptake among swine farm managers in Brittany: age, sex, education level, years of experience, *département*, site size, knowledge of vaccination recommendations, knowledge score, history of influenza, occurrence of influenza on the farm, receipt of a vaccination voucher, colleagues vaccinated, entourage vaccinated, justification for vaccination, fear of vaccination, perceived protection from vaccination, perceived impact of vaccination, perceived benefits of vaccination.

**Supplementary references**

1[. MacDonald NE, SAGE Working Group on Vaccine Hesitancy. Vaccine hesitancy: Definition, scope and determinants. Vaccine. 2015;33:4161‑4. https://doi.org/10.1016/j.vaccine.2015.04.036](https://www.zotero.org/google-docs/?4IuVvz)

2[. Betsch C, Schmid P, Heinemeier D, Korn L, Holtmann C, Böhm R. Beyond confidence: Development of a measure assessing the 5C psychological antecedents of vaccination. PLoS One. 2018;13:e0208601. https://doi.org/10.1371/journal.pone.0208601](https://www.zotero.org/google-docs/?4IuVvz)

**Supplementary** **table S1. Influenza Vaccination Uptake Among Swine Farm Managers: The 7C Psychological Antecedents of Vaccination in three categories– Descriptive and Multivariable Analyses, Brittany, France**

| **7C** | **Variable^a^** | **Total sample**  **N = 735**  **95% CI** | **Unvaccinated**  **n=633**  **95% CI** | **Vaccinated**  **n=102**  **95% CI** | **Fully adjusted multivariable model**  **OR^b^ (95% CI, p-value)** |  |
| --- | --- | --- | --- | --- | --- | --- |
|  |  |  |  |  |  |  |
| **C1 : Calculation** | Benefit/Risk of vaccination, % | | | | |  |
|  | "The flu vaccination provides more benefits than risks for me." | | | | |  |
|  | No | 16.0% [13.7%- 18.6%] | 18.1% [15.5%- 21.0%] | 4.0% [1.6%- 9.9%] | - |  |
|  | Yes | 64.0% [60.7%-67.1%] | 59.4% [55.8%- 62.9%] | 92.2% [85.6%- 95.9%] | 1.92 (0.61-6.04, p=0.267) |  |
|  | Don’t know | 20.0% [16.3%- 21.4%] | 22.5% [18.3%- 24.1%] | 3.8% [1.5%- 9.0%] | 0.36 (0.07-1.81, p=0.216) |  |
| **C2: Social Conformism** | Vaccinated colleagues, % | | | | |  |
|  | "Have people in your professional environment been vaccinated against the flu?" | | | | |  |
|  | No | 50.1% [46.7%-53.4%] | 53.2% [49.6%-56.8%] | 30.8% [23.0%- 39.7%] | - |  |
|  | Yes | 20.8% [18.2%- 23.7%] | 16.8% [14.2%-19.7%] | 45.8% [37.1%- 54.8%] | 2.72 (1.49-4.98, p=0.001) |  |
|  | Don’t know | 29.1% [26.3%-32.3%] | 30.0% [26.8%- 33.4%] | 23.4% [17.4%- 32.4%] | 1.19 (0.61-2.32, p=0.604) |  |
|  | Vaccinated entourage, % | | | | |  |
|  | "Have people in your close circle been vaccinated against the flu?" | | | | |  |
|  | No | 35.5% [32.4%-38.8%] | 38.5% [35.1%-42.1%] | 17.2% [11.7%- 24.7%] | - |  |
|  | Yes | 59.5% [55.9%-62.5%] | 56.3% [52.2%- 59.4% | 79.3% [71.6%- 85.4%] | 2.17 (1.13-4.14, p=0.020) |  |
|  | Don’t know | 5.0% [3.9%-7.0%] | 5.2% [4.0%-7.5%] | 3.5% [1.5%- 8.0%] | 1.21 (0.29-5.10, p=0.793) |  |
| **C3 : Collective responsibility** | Justification for vaccination, % | | | | |  |
|  | "Vaccination of swine farmers is justified to prevent the emergence of new flu viruses." | | | | |  |
|  | No | 23.7% [21.0%-26.6%] | 26.4% [23.4%-29.7%] | 7.0% [3.8%- 12.7%] | - |  |
|  | Yes | 53.7% [50.3%- 57.0%] | 48.8% [45.2%- 52.4%] | 83.8% [76.8%- 89.0% | 2.51 (0.91-6.93, p=0.075) |  |
|  | Don't know | 22.6.2% [20.5%-27.1%] | 24.8% [23.4%-29.8%] | 9.2% [6.1%-15.9%] | 0.83 (0.28-2.49, p=0.739) |  |
| **C4: Confidence in the Vaccine** | Fear of vaccination, % | | | | |  |
|  | "I am afraid of the side effects of the flu vaccine" (R) | | | | |  |
|  | Yes | 36.6% [33.5%-39.9%] | 40.5% [37.0%-44.0%] | 12.8% [7.9%-20.3%] | - |  |
|  | No | 60.2% [56.9%-63.4%] | 56.2% [52.6%-59.7%] | 84.6% [77.0%-90.1%] | 3.05 (1.62-5.74, p=0.001) |  |
|  | Don’t know | 3.2% [2.4%-4.7%] | 3.3% [2.4%- 5.0%] | 2.6% [0.9%- 6.8%] | 6.94 (1.38-34.95, p=0.019) |  |
| **C5: Low Complacency** | Felt protected by the vaccine, % | | | | |  |
|  | "The flu vaccine provides good protection against the disease." | | | | |  |
|  | No | 31.9% [28.9%- 35.1%] | 34.3% [31.0%-37.7%] | 17.4% [11.3%-25.7%] | - |  |
|  | Yes | 48.6% [45.3%- 51.9%] | 44.2% [40.7%- 47.8%] | 75.9% [67.2%-82.8%] | 2.07 (1.06-4.05, p=0.033) |  |
|  | Don’t know | 18.5% [16.5%- 21.8%] | 21.5% [18.2%- 24.1%] | 6.8% [3.6%- 12.5%] | 0.81 (0.29-2.21, p=0.675) |  |
|  | Impact of vaccination, % | | | | |  |
|  | "I am concerned about the impact of flu outbreaks on my farm" | | | | |  |
|  | No | 42.4% [39.2%- 45.7%] | 44.3% [40.7%-47.8%] | 31.0% [23.5%-39.7%] | - |  |
|  | Yes | 55.7% [52.4%- 59.0%] | 53.9% [50.4%-57.5%] | 67.0% [58.2%-74.7%] | 1.19 (0.70-2.03, p=0.527) |  |
|  | Don’t know | 1.8% [1.1%- 3.0%] | 1.8% [1.1%- 3.0%] | 2.0% [0.5%- 7.1%] | 1.70 (0.34-8.49, p=0.515) |  |
| **C6: Confidence in systems** | Hypothetical incitation by OS Porc Brittany, % | | | | |  |
|  | "If the OS Porc encourages you to get vaccinated against the flu, this would..." | | | | |  |
|  | Discourages me | 3.4% [2.4%- 4.9%] | 3.8% [2.6%- 5.4%] | 1.3% [0.2%- 7.4%] | - |  |
|  | Encourages me | 36.4% [33.3% 39.7%] | 32.7% [29.5%- 36.2%] | 59.5% [50.5%- 67.9%] | 5.57 (0.76-40.99, p=0.092) |  |
|  | Have no effect on me | 60.1% [56.8%- 63.3%] | 63.5% [60.0%- 66.9%] | 39.2% [30.9%- 48.2%] | 3.69 (0.50-27.35, p=0.202) |  |
| **C7: Convenience^d^** | Easy access to vaccination, % | | | | |  |
|  | "In practice, is it easy for you to get vaccinated against the flu?" | | | | |  |
|  | No | 8.0% [6.4%-10.0%] | 10.2% [8.3%- 12.6%] | 0.0% [0.0%- 0.0%] | - |  |
|  | Yes | 87.1% [84.7%- 89.1%] | 85.0% [82.3%- 87.3%] | 100.0% [100.0%- 100.0%] | - |  |
|  | Don’t know | 4.9% [3.4%-6.5%] | 4.8% [3.3%- 7.4%] | 0.0% [0.0%- 0.0%] | - |  |

CI = Confidence Interval; OR: Odds Ratio.

*^a^* Note: Percentages are calculated based on the total number of valid responses for each variable

^b^ Odds ratio for being vaccinated in 2023–2024, from the model including age, sex, education level, years of experience, *département*, site size, influenza on the farm during the past six months, knowledge of vaccination recommendation, declaring having received a vaccination voucher, knowledge score and history of “severe” influenza.

^d^ Odds ratio were not calculable due to the absence of vaccinated answering No or Don’t know.

**Supplementary Figure S1. Flow chart of study population**


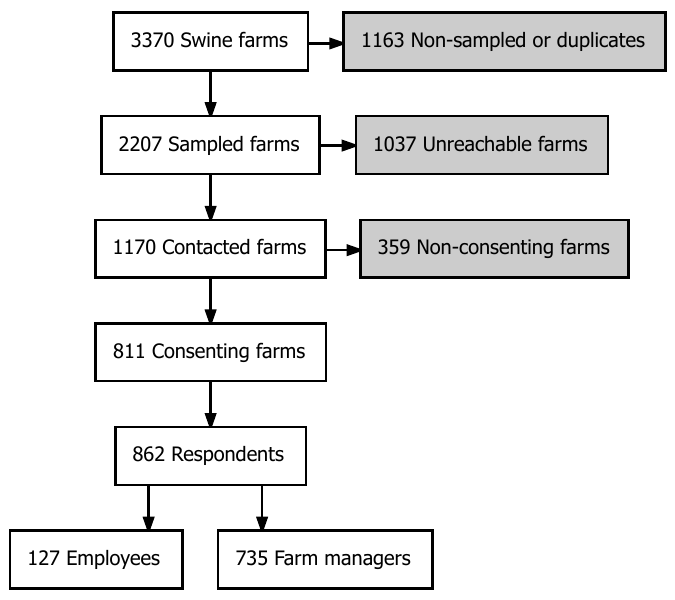

Supplement: Supplementary file 1 — Questionnaire and additional methods [file mmc1.docx]
